# Comparison of non-invasive, scalp-recorded auditory steady-state responses in humans, rhesus monkeys, and common marmosets

**DOI:** 10.1038/s41598-022-13228-8

**Published:** 2022-06-02

**Authors:** Naho Konoike, Haruhiko Iwaoki, Miki Miwa, Honami Sakata, Kosuke Itoh, Katsuki Nakamura

**Affiliations:** 1 Cognitive Neuroscience Section, Primate Research Institute, Kyoto University, Aichi, 484-8506 Japan; 2 Center for Integrated Human Brain Science, Brain Research Institute, University of Niigata, Niigata, Japan

**Keywords:** Neuroscience, Biomarkers, Diseases

## Abstract

Auditory steady-state responses (ASSRs) are basic neural responses used to probe the ability of auditory circuits to produce synchronous activity to repetitive external stimulation. Reduced ASSR has been observed in patients with schizophrenia, especially at 40 Hz. Although ASSR is a translatable biomarker with a potential both in animal models and patients with schizophrenia, little is known about the features of ASSR in monkeys. Herein, we recorded the ASSR from humans, rhesus monkeys, and marmosets using the same method to directly compare the characteristics of ASSRs among the species. We used auditory trains on a wide range of frequencies to investigate the suitable frequency for ASSRs induction, because monkeys usually use stimulus frequency ranges different from humans for vocalization. We found that monkeys and marmosets also show auditory event-related potentials and phase-locking activity in gamma-frequency trains, although the optimal frequency with the best synchronization differed among these species. These results suggest that the ASSR could be a useful translational, cross-species biomarker to examine the generation of gamma-band synchronization in nonhuman primate models of schizophrenia.

## Introduction

Auditory steady-state responses (ASSRs) are basic neural responses that are used to probe the ability of auditory circuits to produce synchronous activity at certain frequencies in response to repetitive external stimulation^1–3^. Typical stimuli that can elicit ASSR are trains of clicks with fixed intervals^4,5^, amplitude-modulated tone^6,7^, or Gaussian tone pulses^8^, particularly in the gamma frequency range (> 30 Hz). After stimulus onset, brain waves rapidly entrain to the frequency and phase of the stimulus. Several neurobiological systems are thought to be involved in the generation of auditory-evoked gamma-band activity^9–14^. Therefore, these contributions suggest that ASSR could be useful as a neurobiological marker of the neural circuit’s functions of auditory-evoked and gamma-range synchronous activity.

Reduced gamma-range ASSR in power or phase-locking has been repeatedly observed in patients with schizophrenia, especially at 40 Hz^11,15–21^. Several studies have reported ASSR abnormalities in rodent models of schizophrenia^10,13,22–24^. However, since the structures and functions of the central nervous system are very different between rodents and primates, a non-human primate model of schizophrenia is of great value in investigation of schizophrenia neuropathology. When evaluating animal models of psychiatric disorders such as schizophrenia, social communication needs to be assessed. However, experiments investigating group socialization in macaque monkeys in a captive environment are challenging. In contrast, marmosets have a great potential as a non-human primate model for psychiatric disorders because of the genetic manipulations variety and the relative breeding and rearing ease, compared to macaque monkeys. Moreover, social behavior of marmosets can be evaluated even in captivity. Therefore, we believe that marmosets represent a promising animal model. To be able to evaluate the ASSR with an appropriate set of stimuli in the future when macaque and marmoset models of schizophrenia are established, the current experiment first examined the response characteristics of normal subjects.

Although several studies have investigated the ASSR with invasive methods^25–27^, to our knowledge no experimental data are available on the characteristics of ASSR from EEG recordings in both macaques and marmosets. Previously, we developed scalp recording methods similar to those applied to humans for rhesus monkeys^28,29^ and common marmosets ^30^. We found that rhesus monkeys and marmosets showed similar auditory event-related potentials (ERPs) to humans^29,30^. To investigate whether the same frequency range of stimuli as used in human studies is appropriate for ASSRs in non-human primates, we recorded the ASSRs from humans, rhesus monkeys, and common marmosets. The same non-invasive scalp-recording method was used and consisted of 1-ms click trains with 30, 40, and 80 Hz, which are used in human experiments^31^, and compared the characteristics of ASSRs among the three species.

Audiograms show that lower-frequency limits are about 32 Hz in both humans and macaques^32–35^, whereas those of common marmosets is about 125 Hz^35–37^. These different hearing abilities among the three species may indicate that the characteristics of ASSR in macaques and marmosets may differ from those in healthy humans. Therefore, we used a wider-range of frequencies to investigate the characteristics of ASSRs in non-human primates.

## Materials and methods

### Subjects and participants

In this experiment, we used five adult common marmosets (*Callithrix jacchus*, two female and three male, 5–8 years old, 272–342 g) and five young adult rhesus monkeys (*Macaca mulatta*, five female, 6–12 years old, 5.2–7.2 kg) for EEG recordings. All marmosets and monkeys were born and housed in temperature-controlled colonies at the Primate Research Institute, Kyoto University. They were maintained on a standard 12-h light/dark cycle. The marmosets and monkeys were fed monkey chow and supplements, such as apples and sweet potatoes. Water was available ad libitum. Experiments were conducted in a sound-attenuated box placed in an experimental room. All experiments for marmosets and monkeys were approved by the Animal Experimentation Committee of the Primate Research Institute of Kyoto University (No. 2019–024, 2020–089 for the scalp electroencephalogram (EEG) recording, No. 2019–025, 2020–111 for the electrocorticography (ECoG) recording) and were conducted in accordance with the Guide for Care and Use of Laboratory Primates published by the Primate Research Institute, Kyoto University, and ARRIVE guidelines (https://arriveguidelines.org/). For human experiments, five healthy Japanese young adult volunteers participated (four female and one male, 24–27 years old). All participants were right-handed and had no neurological or psychiatric history. They were recruited from the Primate Research Institute, and written informed consent was obtained from each participant. All experiments were performed in accordance with the Declarations of Helsinki and the guidelines approved by Kyoto University. This study was approved by the Human Research Ethics Committee of the Primate Research Institute (No. 2019–09).

### Stimulus

The auditory stimuli were generated by a custom script written in MATLAB (MathWorks Inc., Natick, USA) and saved as auditory files (16-bit, 48 kHz). The stimuli were played on a computer using Audacity (https://www.audacityteam.org/). We used a sound interface (sound AV amplifier DSP-AX459, YAMAHA, JAPAN) for the human experiments and sound card (sound blaster Audigy FX, Creative Technology, JAPN) for the animal experiments. For the standard condition, each train contains 1-ms clicks at one of three frequencies (30, 40, or 80 Hz). The duration of each train was 500 ms, and the inter-train interval (ITI; 501–1100 ms from stimulus onset) was 600 ms. Three possible frequencies were presented in a pseudorandom manner. Each block contained 250 trains for each frequency, with 750 trains in total. For the wideband condition, each train contains 1-ms clicks at one of the 11 frequencies (20 to 120 Hz, 10 Hz step). The duration and ITI were the same as those in the standard condition. The trains of 11 possible frequencies were presented in a pseudorandom manner. Each block contained 100 trains for each frequency: 1100 trains in total. Each subject performed two blocks of the standard condition and three blocks of the wideband condition. Each recording block took approximately 14 min for the standard condition and 18 min for the wideband condition. The ITIs were jittered in 8.3 ms (a 1/2 cycle of 60 Hz), meaning that if power line noise at 60 Hz was contaminated, the averaged noise was canceled out because the phase was shifted from trial to trial.

### Apparatus

Prior to EEG recording, marmosets and monkeys underwent a head-shaving procedure under anesthesia and the preparation of head-fitting masks. Each marmoset received an intramuscular injection of alfaxalone (Alfaxaone® 4 or 5 mg/kg, Meiji Seika Pharma) and atropine 0.05 mg/kg. They were supplied with 1:1 oxygen–air mixture (1 L/min) through the mask, and pulse rate and SpO_2_ levels were monitored using pulse oximeters. Each monkey was anesthetized using intramuscular injection of ketamine (Ketalar® 2.5 mg/kg, Daiichi Sankyo Propharma) and medetomidine (0.1 mg/kg). After the procedures were done, the anesthesia was antagonized with atipamezole (0.5 mg/kg, Orion Pharma).

During the experiment, the marmoset or monkey was seated in a primate chair and its head was restricted with a uni-frame thermoplastic mask (Toyo Medic, Tokyo, Japan) in a sound-attenuated room. The stimuli were delivered by a speaker placed 30 cm from the head and controlled at 65–75 dB sound level, measured in a near position of the ear. For the human experiments, the participant was seated in a chair and listened to the same stimuli presented in the animal experiments via a speaker placed 90 cm from the head.

### Data recording and analysis

**Figure 1:**
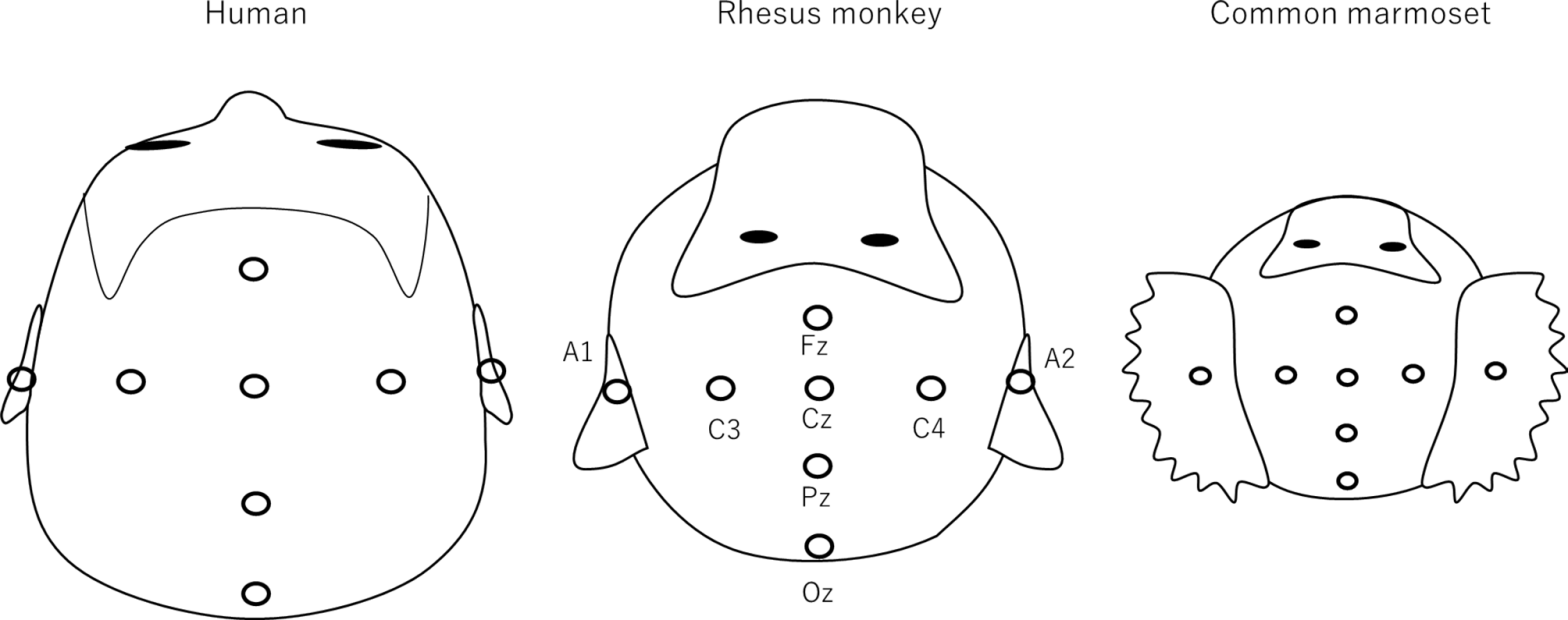
Location of electrodes in humans (left), rhesus monkeys (middle), and common marmosets (right). According to the International 10–20 system, the electrodes were placed with Fz, Cz, Pz, Oz, A1, and A2 in the same locations across all subjects, regardless of the species. The ground electrode was set at C3 or C4.

Silver electrodes with a diameter of 10 mm (NE-136A, Nihon Koden, Tokyo, Japan), 4 mm (UL-3010, Unique Medical, Tokyo, Japan), and 10 mm (C22-834, Technomed, Maastricht-Airport, Netherlands) were placed on the scalp and auricles of the rhesus monkeys, marmosets, and human participants, respectively. For all experiments, EEGs were recorded with six channels (Fz, Cz, Pz, Oz, A1, and A2) according to the International 10–20 System (Fig. 1) with the inion, nasion, and bilateral preauricular points as anatomical landmarks. The C3 or C4 electrode was used as a ground. During the experiment, the electrode impedance was kept below 5 kΩ using an electrode gel. The EEGs were amplified, band-pass filtered (0.016–250 Hz), and sampled at 1000 Hz using BrainAmp (Brain Products, Munich, Germany).

The post-processing of the EEG data was performed using EEGLAB^38^ and ERPLAB^39^. The EEG data were re-referenced to the linked ear reference and high-pass filtered (2 Hz). Then, epochs from 100 ms before to 1000 ms after stimulus onset were created. The data were baseline-corrected to the mean of the 100-ms pre-stimulus period, artifacts were removed using a rejection criterion of ± 150 μV, and averaged for each subject. Finally, group-averaged waveforms were obtained by averaging the ASSR of five subjects for each species. Event-related spectral perturbation (ERSP) was calculated to visualize mean event-related change in spectral power over time in a broad frequency using the formula (1). $F_{k} \left( {f,t} \right)$ is the spectral estimate of trial *k* at frequency *f* and time *t*.

1 $$ERSP\left( {f,t} \right) = \mathop \sum \limits_{k = 1}^{n} \left| {F_{k} \left( {f,t} \right)} \right|^{2}$$

Time–frequency decomposition was applied to the activities using sinusoidal wavelet transforms, three cycles in length at the lowest frequency (10 Hz), increasing linearly with frequency up to 32 cycles at the highest frequency (120 Hz).

Inter-trial coherence (ITC) is a measure of phase consistency over trials, corresponding to the phase-locking factor, which was defined previously^40^. ITC takes values between 0 and 1. An ITC value close to 0 reflects high variability of phase angles across trials, whereas a value near 1 indicates perfect synchronization between EEG data and the time-locking event over trials^38^.

2 $$ITC\left( {f,t} \right) = \frac{1}{n}\mathop \sum \limits_{k = 1}^{n} \frac{{F_{k} \left( {f,t} \right)}}{{\left| {F_{k} \left( {f,t} \right)} \right|}}$$

We calculated the mean ITC values (mITCs) during a 400-ms period of train presentation (101–500 ms from stimulus onset) and a 400-ms period of baseline (601–1000 ms from stimulus onset) in each frequency condition. The mITCs were calculated in the band of 5 Hz lower and higher than the target frequency. Based on the ITC map, brain activity in the initial 100 ms period of train presentation was excluded because the activity may include responses other than ASSR, such as the onset responses, and that in the initial 100 ms period of baseline was also excluded because the offset responses were involved. To investigate whether the mean ICT values during the train presentation were larger than those during baseline, we conducted a one-tailed paired t-test to compare the mITCs between the two periods at each frequency for each species.

In addition, to test the differences of the mITC values among three species, we performed a two-way ANOVA with *stimulus frequencies* as a within-subject factor and *species* as a between-subject factor.

### ECoG data acquisition and analysis in common marmosets

To determine whether filtering some specific components from the brainwave with the skull or subcutaneous tissue produces the frequency characteristics of the ASSR recorded from the scalp, we used two adult female common marmosets (8 and 7 years old, body weights 380 and 400 g) for the ECoG recordings. These marmosets were not used for EEG recording. The animals were anesthetized with ketamine (3 mg/kg), xylazine (1 mg/kg), and atropine (0.05 mg/kg), and the anesthesia was maintained by inhalation of isoflurane (~ 2%). We implanted a 16-channel probe (E16-500–5-200, NeuroNexus, USA) into the epidural space, and the connector was attached to the skull using steel screws and dental cement. The size of the electrode array was 1.8 mm × 2.0 mm. The electrode array of one marmoset was located in the left primary auditory cortex, and another marmoset was implanted in the left prefrontal cortex. The ground and reference electrodes were placed in the epidural space. During the experiment, the awake marmoset was seated in a primate chair and its head was not restricted. ECoG data were recorded at a sampling rate of 1 kHz using a neural recording data acquisition system (OmniPlex, Plexon, Dallas, USA). The experimental apparatus and stimuli were the same as those used for scalp recording. We recorded the neural data during the two sessions of ASSR on a day for both marmosets. The procedures and parameters of analysis of the ECoG data were the same as those of the scalp EEG data described above.

## Results

### Scalp EEG recording

**Figure 2:**
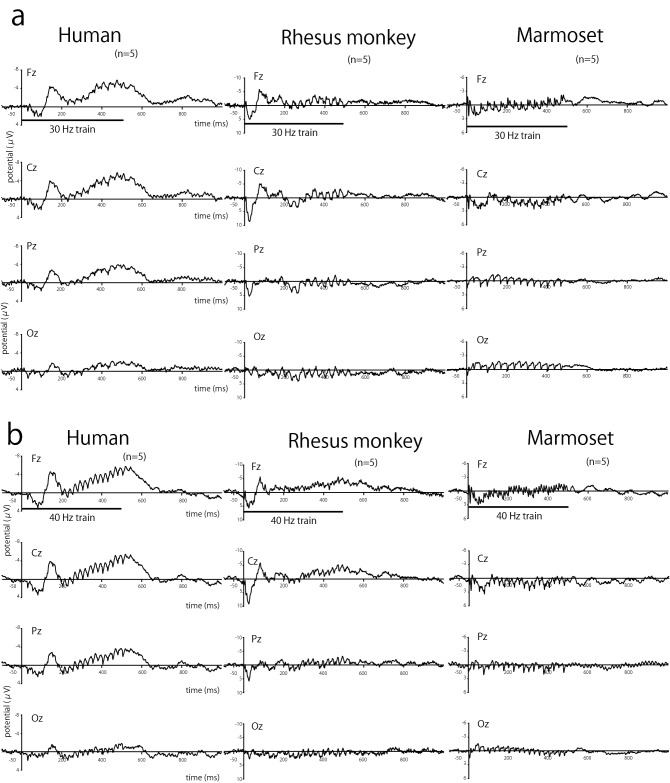

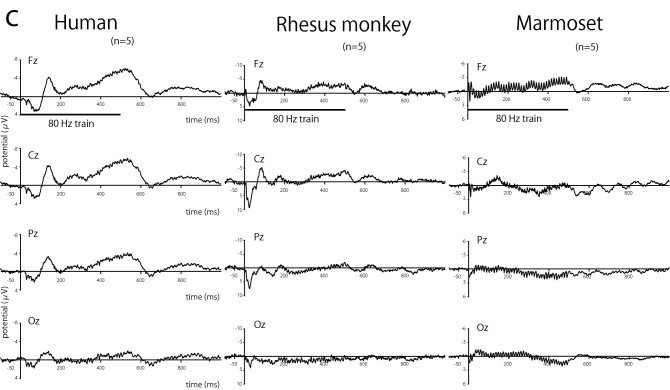
Grand-averaged event-related potentials (ERPs) to (**a**) 30 Hz, (**b**) 40 Hz, and (**c**) 80 Hz click trains for humans (left), rhesus monkeys (middle), and common marmosets (right column; n = 5). The vertical bar indicates the period for the train presentation.

We recorded scalp EEGs from humans, rhesus monkeys, and common marmosets. Figure 2 shows the grand-averaged ERPs to 30 (Fig. 2a), 40 (Fig. 2b), and 80 Hz (Fig. 2c) click trains for all three species. After the onset of train presentation, ERP showed periodic waves of the same frequency as the click train in all species. For humans, the periodicity is prominent in the 40-Hz click trains compared to the 30- and 80-Hz trains. As shown in Fig. 2b, the periodic waves continued during the train presentation and then disappeared after the train offset. On the other hand, the periodicity is also clear for the 30- and 80-Hz trains for monkeys and marmosets (Fig. 2a and c). For marmosets, the ERP to the 30 Hz train in the Fz electrode shows approximately 13 waves during the initial 500 ms of train presentation. These waves consisted of a 30 Hz ASSR (Fig. 2a). In addition, each wave has a notch, which indicates that the brain responds to harmonics to the train at 60 Hz. Similarly, harmonic activity to the 40 Hz train was observed in the marmoset’s Fz electrode (Fig. 2b). Remarkably, the ERP of marmosets in the Fz electrode shows high synchronization to the 80 Hz train (Fig. 2c).

The response to the onset of the train seemed to differ across the three species. The onset responses were quick and prominent in rhesus monkeys, especially in Fz and Cz, whereas they were modest in humans for all frequencies. For marmosets, the first response was weak, with a very short latency. The peak latency of the first response was the shortest in marmoset (11.1 ± 0.7 ms), intermediate in rhesus monkeys (24.8 ± 1.8 ms), and longest in humans (81.6 ± 11.6 ms). There were significant main effects of species on the peak latency of the first response (two-way ANOVA, *F*_(2,10)_ = 3.49, *p* < 0.001), whereas there was no effect of stimulus frequency (*F*_(2,10)_ = 2.35, *p* = 0.49).

**Figure 3:**
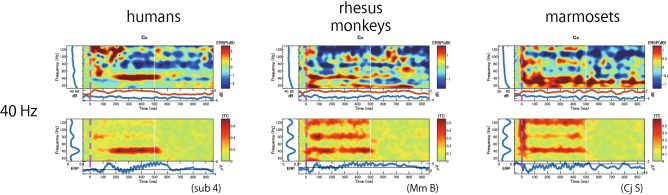
Time–frequency analysis. Example of time–frequency plots of event-related spectral perturbation (ERSP) (upper row in each species) and inter-trial coherence (ITC) (lower row in each species) for the 40 Hz stimulus frequency in humans, rhesus monkeys, and common marmosets. The data was recorded from the Cz electrode. The vertical line of time 0 indicates the beginning of the stimulus, and the vertical white line (on time 500 ms) indicates the end of the stimulus. Horizontal bands were obvious in 40 Hz in both ERSP and ITC maps. In addition to the 40 Hz band, synchronized activity in 80 Hz was also observed as a harmonic response.

The grand-averaged time–frequency plots of ERSP and ITC for 40 Hz stimuli from the Cz electrode in all three species are shown in Fig. 3. The evoked responses were normalized based on a pre-stimulus baseline (− 200 to 0 ms from stimulus onset). The ERSP plots show a remarkable increase in spectral power at 40 Hz during the 40 Hz train presentation for humans. Similarly, the ITC plot reveals that the horizontal band at 40 Hz, indicating high synchronization to the train stimulus, was prominent. These bands promptly disappeared when the presentation of stimuli was stopped 500 ms after stimulus onset. Increasing spectral power and high synchronization at 40 Hz during stimulus presentation were also observed in rhesus monkeys and marmosets. Unlike in humans, synchronization at 80 and 120 Hz, in addition to 40 Hz, was clearly observed in the ITC maps of rhesus monkeys and marmosets. These results indicate that the brain responses to harmonics of the train were obvious for rhesus monkeys and marmosets.

**Figure 4:**
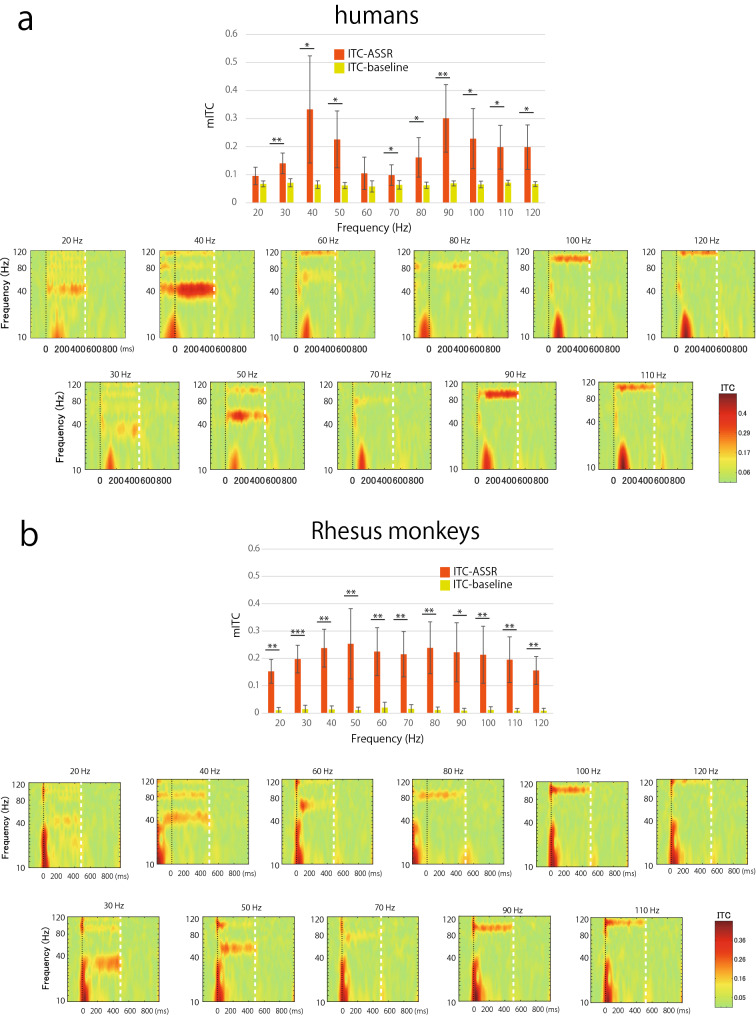

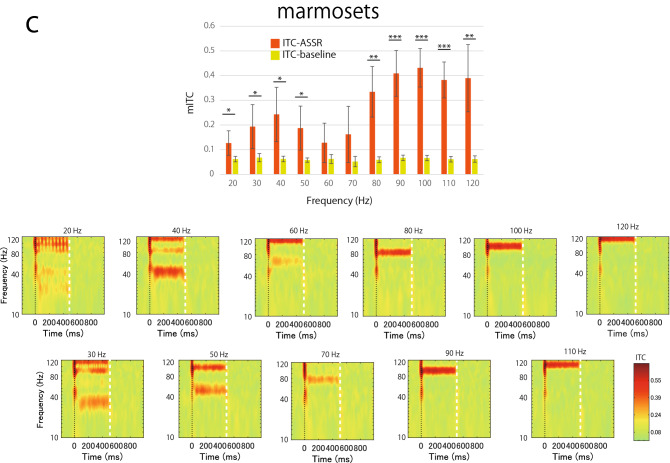
Mean inter-trial coherence (mITC) values and ITC maps in each stimulus frequency in (**a**) humans (**b**) rhesus monkeys, and (**c**) marmosets (n = 5). All data were recorded from the Cz electrode. (Upper panel) Orange bars indicate the mean ITC values (mITCs) during train presentation (ITC-ASSR), and yellow-green bars indicate those during baseline (ITC-baseline) in each stimulus frequency. Horizontal lines and asterisks indicating significant differences between ASSR and baseline (* *p* < 0.05;** *p* < 0.01; *** *p* < 0.001). Error bars indicate the standard deviations of the mean. (Lower panel) Averaged ITC maps in each stimulus frequency (n = 5). The vertical line of time 0 indicates the beginning of the stimulus, and the vertical white line (on time 500 ms) indicates the end of the stimulus.

To evaluate the phase-locking activity synchronized with the repetitive stimuli, the mITCs were calculated and compared during train presentation and those during baseline period under the wideband condition. Figure 4 shows the mITCs calculated from the Cz electrode and time–frequency analysis of ITC for each frequency of stimulus in humans (Fig. 4a), rhesus monkeys (Fig. 4b), and common marmosets (Fig. 4c). For humans, the mITCs during train presentation were significantly larger than those during baseline period for almost all frequencies (*P* < 0.05), except for the 20 Hz (*t*(4) = 1.36, *P* = 0.12) and 60 Hz (*t*(4) = 1.99, *P* = 0.06) trains. In addition, another peak was observed at 90 Hz (*t*(4) = 4.28, *P* = 0.006), showing a bimodal form with high synchronization. The ITC map for each stimulus frequency shows phase-locking activity at the stimulus frequency, as shown by the results for 40 Hz trains in Fig. 3. On the other hand, for rhesus monkeys, the mITCs during the train presentation were significantly larger than those during baseline period for all frequencies with no prominent preference for a specific frequency (*P* < 0.01). Although the mITCs were greatest at 50 Hz (*t*(4) = 4.09, *P* = 0.007) and 80 Hz (*t*(4) = 4.54, *P* = 0.005), there were only slight differences in the mITCs. For marmosets, the mITCs during the train presentation were significantly larger than those during baseline period for almost all frequencies (*P* < 0.05), except for the 60 Hz (*t*(4) = 1.21, *P* = 0.15) and 70 Hz (*t*(4) = 1.95, *P* = 0.06) stimulation. The bimodal pattern of the mITCs with one peak at 40 Hz (*t*(4) = 3.26, *P* = 0.02) and another at 100 Hz (*t*(4) = 8.67, *P* = 0.0005) was similar to that in humans, but the marmosets showed higher synchronization at higher gamma frequencies (more than 80 Hz).

The differences of the mITC were examined by a two-way ANOVA with *stimulus frequencies* as a within-subject factor and *species* as a between-subject factor. No significant main effects of both *stimulus frequencies* and *species* were observed in the mITCs (*F*(10, 120) = 3.79, *P* = 0.15 and *F*(2, 12) = 1.71, *P* = 0.22, respectively). The interaction between stimulus frequencies and species was statistically significant (*F*(20, 120) = 6.741, *P* = 0.04). Since the interaction was significant, we concluded that there may be a species difference in the frequency response characteristics. However, a post-hoc analysis showed no significant difference. This may be due to the small sample size. Therefore, we performed a nonparametric test with correction for multiple comparisons to examine the mITC differences by stimulus frequency on each species. We found that a significant difference among stimulus frequencies in humans (Friedman test, *S*(10) = 20.15, *P* = 0.03) and marmosets (*S*(10) = 38.55, *P* < 0.001), however, no significant difference in rhesus monkeys (*S* (10) = 8.80, *P* = 0.55). A post-hoc analysis with multiple comparisons (Bonferroni) revealed that a significant difference of mITC between 20 and 90 Hz (*P* = 0.047) in humans, and between 20 and 100 Hz (*P* = 0.02), 60 and 90 Hz (0.047), 60 and 100 Hz (*P* = 0.002), 60 and 110 Hz (*P* = 0.011), and 60 and 120 Hz (*P* = 0.016) in marmosets. These data suggest that the ASSRs to the 20–120 Hz stimulation were observed in common marmosets and rhesus monkeys like humans, but they showed different patterns of ASSR from low to high gamma frequencies.

In addition to the phase-locking activity, a low-frequency transient response of approximately 10–40 Hz immediately after stimulus onset was observed in humans and rhesus monkeys (ITC maps in Fig. 4). In marmosets, a similar component is approximately 40–80 Hz. These components in the ICT maps correspond to the neural responses to the onset of the train observed in the averaged ERP (Fig. 2). Furthermore, low-frequency responses around the train offset were observed in ITC maps with stimulus frequencies above 60 Hz, especially for human and rhesus monkeys. This seems to be a neural response to the stimulus offset (ITC maps in Fig. 4).

### ECoG recording

**Figure 5:**
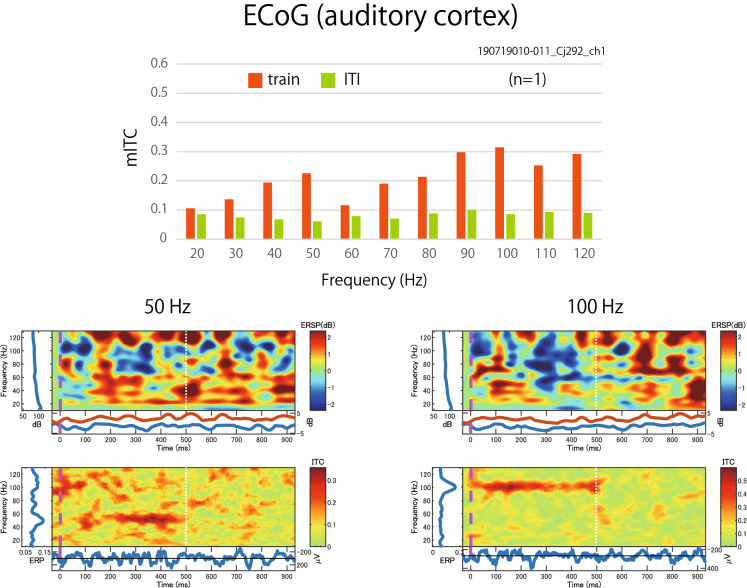
The mean Inter-trial coherence (mITC) values in each stimulus frequency were calculated by electrocorticography (ECoG) data from the auditory cortex of one marmoset (Cj292). Orange bars indicate the mean ITC values (mITCs) during train presentation, and light green bars indicate those during inter-train intervals (ITIs). The time–frequency plots of event-related spectral perturbation (ERSP) (upper row) and inter-trial coherence (ITC) (lower row) for the 50 Hz and 100 Hz stimulus frequency in common marmosets. The data was recorded from Ch1. The vertical purple line of time 0 indicates the beginning of the train, and the vertical white line (on time 500 ms) indicates the end of the train.

We also recorded the ASSR using ECoG from the auditory cortex and the prefrontal cortex of other two common marmosets using the same stimuli and compared it with the results of scalp recordings. Figure 5 shows the mITCs (upper panel), which were calculated from the ECoG data from the primary auditory cortex. In both the auditory cortex and prefrontal cortex (not shown), the mITCs for all frequencies showed similar bimodal patterns. The frequencies of the peaks were slightly different, but there was one peak at low frequencies (40–50 Hz) and another peak at high frequencies (above 80 Hz). The time–frequency maps of ERSP and ITC for 50 and 100 Hz stimuli are shown in Fig. 5. A higher phase-locking activity was observed in high-gamma band stimuli. These features are essentially the same as those of scalp data.

## Discussion

We recorded the ASSR synchronized to gamma-band stimuli from humans, rhesus monkeys, and common marmosets using the same non-invasive method. The ERPs at each stimulus frequency showed periodic activity synchronized with the stimulus frequency. The spectrogram also showed a peak of power consistent with each stimulus frequency. In addition, the ITC, which reflects the temporal and spectral synchronization and captures the consistency in phase alignment of neuronal activity that is elicited by auditory stimuli, was larger during the stimulus presentation period than during baseline period without stimulus in all three species. These results indicate that ASSRs were present in rhesus monkeys and common marmosets as well as in humans using the same scalp recording technique. Most studies of ERPs in monkeys have used invasive techniques, including brain surgery, to implant electrodes inside the skull^41–45^. In recent years, several studies have reported the use of non-invasive methods for recording electroencephalography (EEG) in monkeys^46–50^. We have previously demonstrated the non-invasive scalp recording of cortical auditory evoked potentials (CAEPs) in alert rhesus monkeys^28,29^ and common marmosets^30^. Such non-invasive scalp recordings of non-human primates are promising because they can be directly compared with the non-invasive EEG measurements routinely performed in humans. In addition, noninvasive EEG measurements allow EEG data to be obtained without surgically damaging valuable and limited numbers of primate models of psychiatric diseases. In the current study, we applied this method to the ASSR, and, to the best of our knowledge, this is the first report of ASSR in non-human primates using a non-invasive method. The ASSRs for the gamma frequencies are thought to be generated from the auditory cortex and subcortical regions^21,51–53^. A neurophysiological study has reported that the neurons in the auditory cortex of anesthetized monkeys synchronize their firing rate to the individual click in trains up to 100 Hz^42^. However, higher frequencies are thought to originate from the brainstem areas^53^. The inferior colliculus may play an especially important role in the generation of 80-Hz responses. In the current study, we recorded higher synchronized activity to trains with high frequencies, particularly in marmosets. The marmoset’s head is small, so that the neuronal activity in the inferior colliculus may be easily captured by electrodes on their scalp. Nevertheless, we demonstrated that human participants also showed significantly high phase-locking activity at high gamma trains. The results suggest an important role of ASSR in the high-gamma stimuli.

### Comparison among three species

We found a difference in neuronal entrainment to auditory trains with 10–120 Hz frequency among humans, rhesus monkeys, and common marmosets. Humans and marmosets showed one peak of high synchronization to a 40 Hz train and another peak to high gamma trains (90 and 100 Hz), which is a similar bimodal pattern. For the response to high gamma trains (> 80 Hz), the phase-locking activity was large in marmosets. On the other hand, rhesus monkeys showed high synchronization to all frequencies, but no obvious preference for specific stimuli. There are several possible reasons for these differences among primate species.

These three species differ in their characteristics of basic auditory function—in their audible frequency ranges, most sensitive parts of the audiogram^35,37,54–57^, and frequency content of their vocalizations^58–61^. These differences may partly explain our current findings indicating that the ITC of the response to stimuli at 40 Hz was the largest in healthy humans, whereas the maximal coherence in rhesus monkeys was at 50 Hz and 100 Hz in marmosets. Alternatively, the frequency shift may also be dependent on brain volume^62^ and the number of neurons^63^ in each species.

### Harmonics

Harmonic activities were observed in the time–frequency map during train presentation at lower frequencies (Figs. 3 and 4). This is because we used the click train as a stimulus that has harmonic spectral cues and activates several tonotopic areas corresponding to each frequency, whereas the modulation frequency of amplitude-modulated tones has only one peak at the stimulus frequency^64^. These harmonic activities were more prominent in marmosets than in rhesus monkeys and humans. A previous study has reported that macaque monkeys showed no preference for harmonic sounds^65^. In contrast, neurons that sensitive to the combination of temporal envelope and spectral cues were recorded in marmoset auditory cortex^66^. Furthermore, the resolvability of harmonics in marmosets is different from that in humans^67^. These results are consistent with our results showing strong harmonic activity in marmosets. Harmonic activity in marmosets may be a clue to the evolution of human speech.

### Comparison with ECoG data

We found that the ASSRs recorded from the scalp EEG showed the highest synchrony with the auditory stimuli at high gamma frequency, and the result was the same as the ECoG data recorded from the primary auditory cortex (Fig. 5) and the prefrontal cortex (not shown). Scalp EEG can detect signals when the cortex is active in a synchronized manner over 10 cm^2^ in humans^68,69^, whereas the spatial resolution of the ECoG was higher (approximately < 5 mm^2^ in humans)^68^. The activity at a high frequency was difficult to record on the skull because the signals were obscured by electrical artifacts and scalp EMG^70,71^, and the skull acts as a low-pass filter. Based on these characteristics of EEG recordings, we showed the results recorded by ECoG to demonstrate that the features of the ASSRs recorded from EEG are not affected by distance from the signal source or attenuation of certain frequencies. The results suggest that the characteristics of ASSR obtained from scalp recording are sustainable.

### Transient responses

The measurement of ASSRs allows us to evaluate not only the synchronous activation of neuronal ensembles but also the transient response to auditory stimuli^19^. We found CAEP as a response to the onset of each train. The response to the first stimulus was more apparent than the responses to the other stimuli, but it was partly obscured by the periodic waveform of the ASSR. The first transient response has a positive peak and a robust negative peak; therefore, the response is thought to be homologous to that of P1 and N1 in humans. The responses were prominent in Fz and Cz, reflecting that the CAEP originates in the first stages of temporal integration of thalamocortical inputs to the cerebrum and following a higher level of auditory processing in the auditory cortex. The peak latencies of the first positive responses were longest in humans (81.6 ± 11.6 ms), intermediate in rhesus monkeys (24.8 ± 1.8 ms), and shortest in marmosets (difficult to define). These data are consistent with previous reports^72–74^.

### As a translatable biomarker

A reduction of ASSRs to gamma-band frequencies in power or phase-locking activity has been repeatedly observed in patients with schizophrenia, especially ASSR at 40 Hz^12,15,19,31,75–78^. The generation of gamma-band synchronization has been suggested to be dependent on the network of GABAergic interneurons that act as pacemakers and produce rhythmic inhibitory postsynaptic potentials in pyramidal neurons^9,14,79^. In addition to GABAergic dysfunction, loss of N-methyl-D-aspartic acid (NMDA) receptor function in inhibitory parvalbumin interneurons leads to abnormal hyperactivity of excitatory neurons, which results in impaired synchronization to external stimuli. Thus, ASSR reduction in schizophrenia reflects the dysfunction of GABAergic and/or NMDA receptors. In the current experiment, we demonstrate that ASSRs are available in non-human primates to evaluate gamma-band synchronization to external stimuli by the scalp recording method. These methods enable us to use ASSR as a translatable biomarker between non-human primate models of schizophrenia and patients. Many EEG studies in patients with schizophrenia have focused on ASSR to lower-gamma frequencies^11,15,17,18,75^. However, as described above, distinct neural networks have been suggested to be involved in low- and high-gamma responses^53^. A magnetoencephalography (MEG) study has reported that the power of ASSRs at 40 Hz and 80 Hz was decreased in patients with schizophrenia^19^. Furthermore, another MEG study reported a reduction in power and phase-locking activity at 80 Hz ASSR in patients with schizophrenia, and the lower 80 Hz ASSR was related to hallucination but not related to the reduction of the power of ASSR at 20 or 30 Hz^21^. These results suggest that abnormal high-gamma synchronization is related to positive symptoms of schizophrenia, and the dysfunction of ASSR at high-gamma frequencies may be more involved in the pathogenesis of schizophrenia than previously thought. In the future, it may be possible to examine abnormalities in different brain regions and neural networks in patients with schizophrenia by measuring and analyzing the ASSR at low and high gamma frequencies.

In summary, at least for ASSR recording of marmosets, non-invasive recording from the scalp is considered sufficient because the auditory response characteristics are not altered when compared to ECoG, which is an invasive intracranial implantation of electrodes. This method allows us to directly compare brain activity as a potential biomarker, including the ASSR, P300, N50, and mismatch negativity, for neuropsychiatric diseases between humans and non-human primate models. Further experiments are required to confirm this hypothesis.

## Supplementary Information

Supplementary Information.

## Data Availability

The data that support the findings of this study are available from the corresponding author upon reasonable request.
