# Supplementary figures and images for: Comparison of non-invasive, scalp-recorded auditory steady-state responses in humans, rhesus monkeys, and common marmosets
Source: Sci Rep. 2022 Jun 2;12:9210. doi: 10.1038/s41598-022-13228-8 (PMC9163194; doi:10.1038/s41598-022-13228-8)

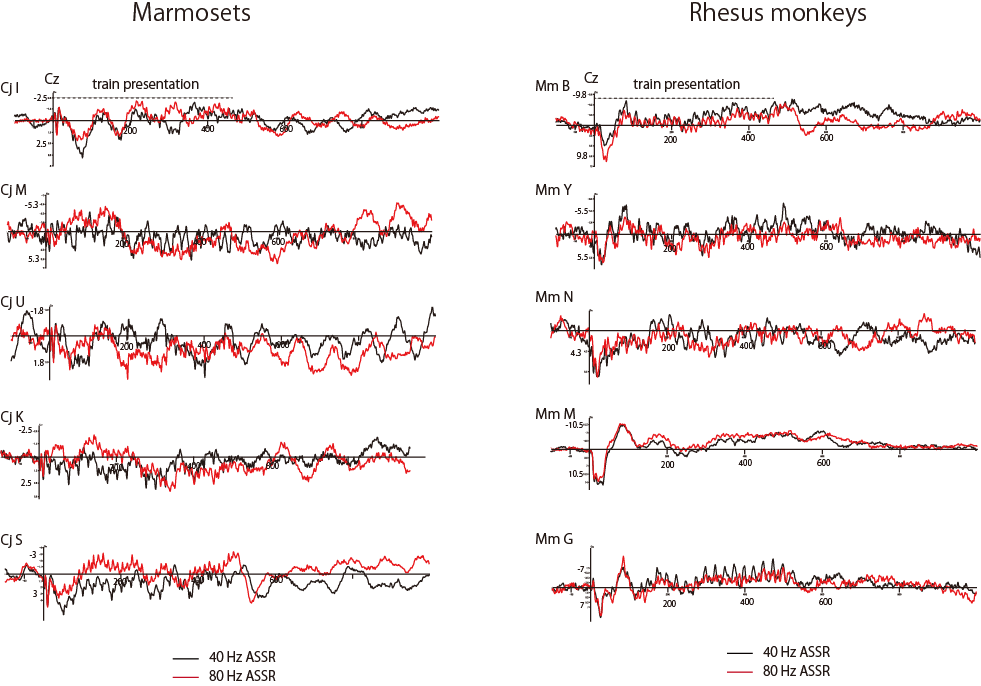


Supplementary Figure 1

Supplement: Supplementary file 1 — Supplementary Information. [file 41598_2022_13228_MOESM1_ESM.docx]
